# Impact of the ABCDE triage on the number of patient visits to the emergency department

**DOI:** 10.1186/1471-227X-10-12

**Published:** 2010-06-03

**Authors:** Jarmo Kantonen, Johanna Kaartinen, Juho Mattila, Ricardo Menezes, Mia Malmila, Maaret Castren, Timo Kauppila

**Affiliations:** 1 City of Vantaa, Finland; 2 Attendo-Medone LTD, Finland; 3 Peijas Hospital, Helsinki University Central, Hospital, Finland; 4 Helsinki University Central Hospital, Helskinki, Finland; 5 Emergency unit project, Jorvi Hospital, Puolarmetsä Hospital (City of Espoo), Finland; 6 Karolinska Institutet, Department of clinical Sciences and Education, Södersjukhuset, Stockholm, Sweden; 7 Network of Academic Health Centres, Departments of General Practice and National Public Health, University of Helsinki, Finland; 8 Department of Primary Healthcare, Institute of Clinical Medicine, and Department of National Public Health, University of Helsinki, Finland; 9 Department of National Public Health, University of Helsinki, Finland

## Abstract

**Background:**

Many Finnish emergency departments (ED) serve both primary and secondary health care patients and are therefore referred to as combined emergency departments. Primary care specialists are responsible for the initial assessment and treatment. They, thereby, also regulate referral and access to tertiary care. Primary health care EDs are easy for the public to access, leading to non-acute patient visits to the emergency department. This has caused increased queues and unnecessary difficulties in providing immediate treatment for those patients who need it the most.

**Methods:**

A face-to-face triage system based on the letters A (patient directly to secondary care), B (to be examined within 10 min), C (to be examined within 1 h), D (to be examined within 2 h) and E (no need for immediate treatment) for assessing the urgency of patients' treatment needs was applied in the main ED in the City of Vantaa, Finland (Peijas Hospital) as an attempt to provide immediate treatment for the most acute patients. The first step was an initial patient assessment by a health care professional (triage nurse). If the patient was not considered to be in need of immediate care (i.e. A-D) he was allocated to group E and examined after the more urgent patients were treated. The introduction of this triage system was combined with information to the public on the "correct" use of emergency services. The primary aim of this study was to assess whether the flow of patients was changed by implementing the ABCDE-triage system in the combined ED. To study the effect of the intervention on patient flow, numbers monthly visits to doctors were recorded before and after intervention in Peijas ED and, simultaneously, in control EDs (Myyrmäki in Vantaa, Jorvi and Puolarmetsä in Espoo). To study does the implementation of the triage system redirect patients to other health services, numbers of monthly visits to doctors were also scored in the private health care and public office hour services of Vantaa primary care.

**Results:**

The number of patient visits to a primary care doctor in 2004 decreased by up to eight percent (340 visits/month) as compared to the previous year in the Peijas ED after implementation of the ABCDE-triage system. Simultaneously, doctor visits in tertiary health care ED increased by ten percent (125 visits/month). ABCDE-triage was not associated with a subsequent increase in the number of patient visits in the private health care or office hour services. The number of ED visits in the City of Espoo, used as a control where no triage was applied, remained unchanged.

**Conclusions:**

The present ABCDE-triage system combined with public guidance may reduce patient visits to primary health care EDs but not to the tertiary health care EDs.

## Background

Low acuity visits in the ED may cause significant problems since they consume resources that should be allocated for high acuity patients [1-4]. Triage has, in part, been developed in order to allocate resources [3,4]. Strategies aimed at diverting non-urgent patients by using triage did not seem to improve access of more urgent patients in a Canadian tertiary health care ED (university hospital). This may be explained by the observation that the probability of a patient to have a severe and/or life threatening was high and non-acute patients represented only a small fraction of the patient flow [3]. There is some data from tertiary health care systems suggesting that team-triage may reduce the time to doctor, time to radiology and the length of stay in the ED [5]. Experienced doctor-nurse triage teams have been reported to be an effective way of shortening the waiting time in the ED, irrespectively of the urgency of the condition [5].

In Finland EDs are funded by the public health system and are non profit. Emergency services in Finland have been provided by both hospitals and health centres since the 1970 s. After hours services in health centres are run by primary health care staff and GPs while the EDs of the tertiary hospitals are run by different medical specialities. Primary care out-of-hours units were increasingly incorporated into hospital emergency units due to centralization at the end of the 20th century. These EDs came to be known as 'combined emergency departments' [6]. GPs are responsible for the initial assessment and treatment in the EDs, thereby regulating access to the acute tertiary health care. One argument for this centralization is that a considerable number of patients needing acute care, also require hospital treatment, tests performed in hospital and medical attention from specialists [6]. After hours services were used less when the office hours of the public primary health care centres were improved in the 1990's by the so called personal doctor system[6]. Decreased use of EDs indicated that a smoothly running service during office hours reduced the demand for after hours services [6]. This is observed to be a general trend when the quality of daytime primary care is adequate [7]. As a complementary profit driven system, there has been a well equipped private primary health care which is, however, expensive to use. Patients choosing this system cover the expenses by using private money or insurances.

The situation in Finish primary care has recently become worse due to a decreased recruitment of doctors to the public health system. As a consequence, access to daytime services has worsened [6] and EDs are forced to back up the inadequate daytime services in primary, secondary and tertiary care. Easily accessible EDs may also be considered as an extra public service for those who are, for various reasons [4], not willing or able to use daytime services. The EDs are overused and this situation has led to negative patient feedback and increased frustration of the staff [8]. There have been difficulties in the recruitment of doctors and a rapid progression in outsourcing the work of the GPs to agency employees due to the nature of the work and inconvenient working hours, [6,8]. Thereby, the variability of primary care doctors especially for after hours services has been high [6]. It has also been difficult to recruit experienced nursing staff to the emergency system in primary health care. Many stakeholders and organizations are involved in the provision of emergency services making the responsibility for the leadership and the development of the EDs unclear.

Emergency services should be capable of providing quick and effective treatment to patients with acute medical problems. This capability is, however, compromised if the ED is too crowded [9]. Inaccurate assessment at the point of first contact may lead to unnecessary or incorrect treatments and processes. Therefore, organizational attempts to redirect inappropriate patient flow had to be taken. Because GPs are supposed to regulate access to the tertiary health care in combined EDs, changes in triaging patients might alter the patient flow in the entire emergency system.

As an attempt to provide immediate treatment for patients who need it the most, a face-to-face triage system [10] based on letters from A, B, C, D and E for assessing the urgency of patients' treatment needs was applied in the main combined ED in the City of Vantaa, Finland (Peijas Hospital). The primary aim of our study was to determine whether this type of triage system alters the patient flow (monthly number of visits) in the GP driven department (primary health care) and the specialist driven department (tertiary health care) of this type of ED. A secondary aim was to study if triage in the combined ED increases the number of patients in public or private primary health care. We also studied if this triage system would have an impact on emergency referrals from the primary to the tertiary health care.

## Methods

### Sample

This study was performed in Peijas hospital ED which serves as an after hours primary health care service for the City of Vantaa. Vantaa has a population of 182,000 inhabitants. Since tertiary health care is also present in Peijas, it is defined as a combined ED. It is equipped with out-of-hours laboratory and X-ray facilities. The other ED in Vantaa, Myyrmäki ED, resembles a more traditional Finish primary health care out-of-hours unit where no specialist care is provided and the laboratory and X-ray facilities are available only during office-hours.

Puolarmetsä and Jorvi, out-of-hours primary health care services for the City of Espoo, a neighbor city to Vantaa with a population of 222 000 inhabitants served as a control. Jorvi is a combined ED, while Puolarmetsä resembles a more traditional Finish primary health care out-of-hours unit.

### Variables

The data was obtained from the electronic health records of Vantaa (Finstar-patient chart system), Espoo (Effica- patient chart system) and Peijas tertiary health care ED (Helsinki University Central Hospital (HUCH) Musti- patient chart system). KELA (The Social Insurance Institution of Finland) provided the data from the private primary health care doctors. The monthly numbers of referrals to Peijas tertiary ED was gathered from the Musti-system. In Vantaa, the follow-up was performed between January 2003 and December 2005. Due to the changes in the electrical patient chart system in Espoo, we failed to obtain data from January to April 2003. The number of monthly visits to doctors was scored in each study department before and after implementation of the ABCDE triage system (1.1. 2004). Thus, we could study the situation before and after the implementation of ABCDE-triage in Peijas ED and compare changes of measured parameters with the Myyrmäki, Puolarmetsä and Jorvi EDs where no triage was applied. No ethical approval was required because this study was made directly from the patient registry without identifying the patients. The registry keeper (health authorities Vantaa, Espoo and HUCH) accorded permission to do the study.

### Intervention

Leaders responsible for the implementation of the intervention (project) were chosen. The project workers analyzed the process and patients in need of special attention were identified based on interviews of health policy specialists. These were elderly people, children and people suffering from mental illness or drug abuse. A discussion was raised in the media around these services and information was delivered both to professionals and the public and, thereby, the impact of introducing the ABCDE-triage tool in emergency services was also enhanced by increasing the publicity about the issue. Guidelines for the staff when performing the triage; changes were enabled by training, and through motivation and encouragement. The general public was informed of the project through the media and the information focused on the transparency of the system. Internet, local print media, radio and bulletins were used. The aim of the project group was to publish as much information as possible related to the changes to keep the population, all organizations associated with the project and the staff fully informed. The objective of this massive information campaign was to guide non-acute patients to appropriate day time services. Feedback was actively gathered both from patients and the staff with questionnaires and interviews. Numbers of visits to doctors and nurses, assessed patients, triage groups, waiting times and diagnoses were frequently assessed. The staff was encouraged to follow the guidelines and provide leaders with useful information. Follow-up meetings were organized in order to discuss the implementation process and problematic patient cases.

ABCDE-triage [10] was performed by an experienced nurse, in the first line of the emergency service, assessing the patients before being attended by the doctor. The patients were triaged subjectively by the nurse as shown in Table 1. From January 2004 to December 2005 the group E-patients were able to stay and wait if they wanted to see a doctor even though the triage nurse had explained to the patient, that his/her case was assessed to group E (non-acute). If the status of the patient altered in the waiting room a re-triage was performed.

**Table 1:** The 5 (five) scale groups from A to E used at Peijas ED.

A	immediate care (for example resuscitation)
B	the patient must be seen by a doctor (usually a specialist) within 10 minutes (acute crises)
C	the patient needs to meet a doctor within 1 hour (severe infections, trauma etc)
D	the patient needs to meet a doctor within 2 hours (minor trauma, less severe infections etc)
E	Not an acute patient, must wait till more urgent patients from groups A-D were treated (non-urgent problems: mild upper respiratory tract infections, mild fever, cough, chronic symptoms in back, pain in ear, mild diarrhoea or vomiting, prolonged general weakness and tiredness)

### Statistical analysis

The triage system was introduced at the beginning of January 2004. The number of monthly patient visits in 2004 and 2005 were compared to the number of patient visits in the respective months of the year 2003 when triage was not yet applied. There were systematic monthly variations in the numbers of doctor visits (see first paragraph of the Results) and, therefore, one-way ANOVA of repeated measurements followed by t-test with the Bonferroni Correction was chosen as the method for statistical analysis [10]. When appropriate, paired t-test was applied.

## Results

The number of monthly visits to doctors differed significantly during day time in Vantaa and Espoo (ANOVA F{11,57} = 30,445, p < 0,001) and in the private sector (ANOVA F{11,60} = 4,763, p < 0.01). July proved to be by far the least frequented month in primary health care of Vantaa and Espoo and in the private sector (p < 0.01).

**Figure 1:**
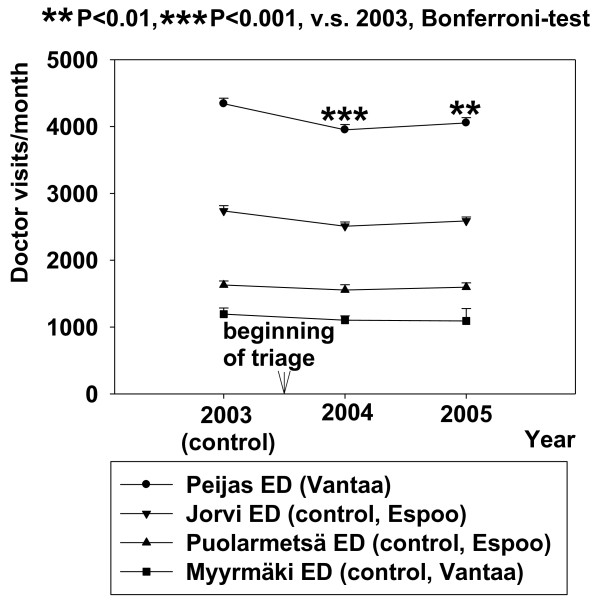
**Effect of triage on doctor visits in Peijas ED, and a comparison with EDs where triage was not applied**. Data are shown before and after triage. Mean ± SE is shown.

**Figure 2:**
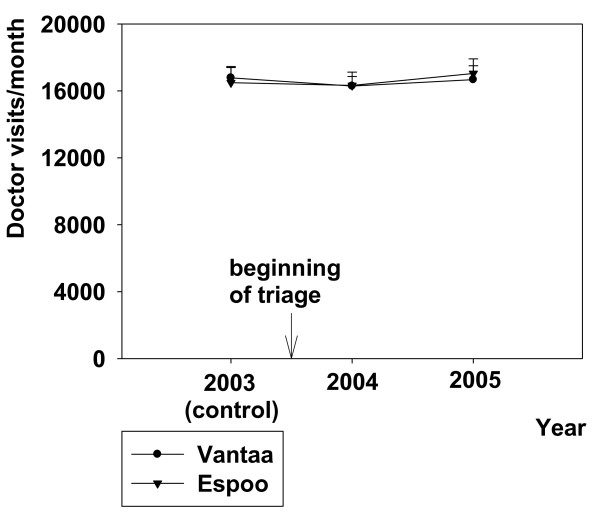
**Effect of triage in Peijas ED on office-hour doctor visits in Vantaa, a comparison with control (Espoo)**. Data are shown before and after triage. Mean ± SE is shown.

The introduction of the ABCDE- triage system decreased the number of the doctor visits (about 340 visits/month) from the numbers of the control year (2003) in primary health care at Peijas ED (RM-ANOVA, F{11,2} = 14,343, p < 0.001) while in control EDs, eg. Jorvi (p = 0.07), Puolarmetsä (p = 0.65) or Myyrmäki (p = 0.52), showed no significant changes (Figure 1). The implication of triage in Peijas ED did not change the number of monthly doctor visits in office hour public services in Vantaa or Espoo (mean; 16300-17000 visits/month, Figure 2).

The patient chart system did not record the triage group of the patients automatically. Therefore only an individual hand-picked sample (March 2004) was available. According to this sample, 6,3% of the patients were triaged to group C, 22,4% to group D and 25.2% to group E. The biggest group contained the most acute patients (A-B) and produced 46.2% of the visits.

**Figure 3:**
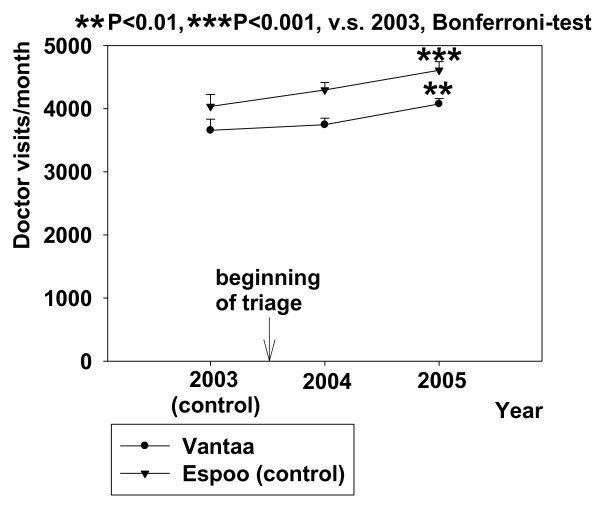
**Effect of triage in Peijas ED (Vantaa) on visits to private sector GPs, and a comparison with Espoo (control)**. Data are shown before and after triage. Mean ± SE is shown.

Doctor visits to the GPs of the private sector in Vantaa increased one year after the beginning of the intervention by about 420 visits/month (at year 2005, RM-ANOVA F{11,2} = 5,581, p < 0.05) while they increased by roughly 570 visits/month in the control city Espoo (at year 2005, RM-ANOVA F{11,2} = 11,695, p < 0.001, Figure 3). There was no change immediately after implementation of triage (year 2004) in either city. The proportional increase in the use of the private sector in the control city Espoo was roughly 15%, almost the same as it was in Vantaa (13%). Altogether, the number of monthly doctor visits in the private sector was higher in Espoo (mean ± SD; 4313 ± 562) than in Vantaa (3826 ± 466, P < 0.001, paired t-test).

**Figure 4:**
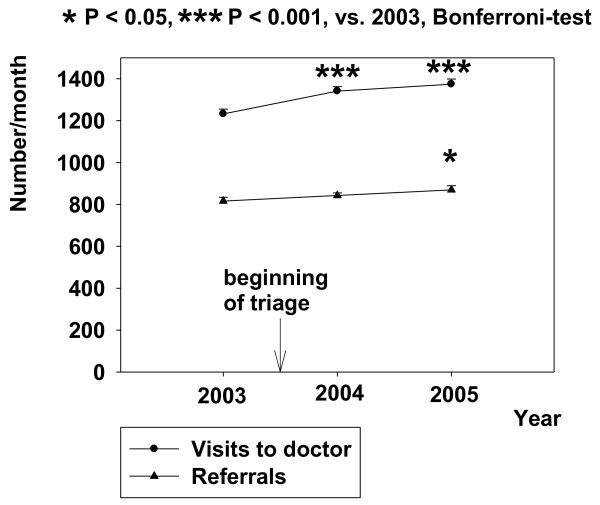
**Effect of triage on visits and referrals to tertiary health care in Peijas ED**. Data are shown before and after triage. Mean ± SE is shown.

In the tertiary health care ED of Peijas hospital (HUCH) implementation of triage in primary health care of the same facility increased use by 125 visits/month immediately during year 2004 (RM-ANOVA F{11,2} = 22,675, p < 0.001) but the number of referrals to the tertiary health care did not increase until year 2005 (RM-ANOVA F{11,2} = 4,129, p < 0.05, Figure 4). The increase was smaller in the number of referrals to tertiary health care ED (e.g. 50 referrals/month) than the increase in the number of visits (e.g. 125 visits/month) to the respective facility.

## Discussion

The implementation of the ABCDE-triage system for assessing the patient acuity at Peijas combined ED reduced the number of patient visits to GPs of the ED by eight percent. Neither the similar type of Jorvi ED in Espoo, nor the more conventional types of primary health care EDs, Myyrmäki in Vantaa or Puolarmetsä in Espoo, resulted in decreased use during the same time period (Figure 1). The observed reduction in GP visits in the ED may partly be due to considerable public debate and the publicity provided by the new system. Patients were, thus, allowed to stay and wait for the service if they so wished. Putatively, some of the patients decided not to request emergency care due to the expected long waiting times and the number of visits to GPs in ED decreased. The population seemed to adapt very quickly to the idea that those who needed help most must go first and those whose need is not urgent should not necessarily visit the ED at all.

GPs in the present system were previously assumed to regulate access to the acute tertiary health care by redirecting the patients and when necessary, to more appropriate health care services. Despite this, use of ABCDE triage in the combined ED with a subsequent decrease in visits to GPs was associated with an immediate ten percent increase in visits to Peijas' tertiary health care ED (Figure 4). In practice, this meant four additional emergency patients to the University hospital every day. Obviously, many of these patients came without referral from the primary health care because there was no subsequent increase in the number of referrals instantly after the beginning of triage in 2004. In a nutshell, triage was performed by primary health care EDs but it was associated with an increased work load of the tertiary health care in the same facility. Altogether, the present finding agrees with the former report of Vertesi [3] which suggested that triage did not enhance activities in the tertiary health care ED. As far as we know, the present type of study is one of the first of this kind. Kuensting studied where the so called out-triaged children with minor health problems end up [11]. As a rule, however, the former studies about use of triage in the ED have concentrated more on changes in internal patient flow [3,5,12-14] than on how the triage alters use of the studied facility and other parts of the health care system. The lack of national standards and guidelines or other governing documents on ED triage may partly be a result of the absence of operational and research attention given to this issue [14].

Overcrowding and excessive delays are a serious problem in urban specialist driven EDs and it is possible that many patients who seek care could be managed in lower acuity settings. Former studies suggested that in some EDs 30% to 50% of visiting patients could be appropriately cared for at their own health center during normal office hours, and if this is true, diverting non-urgent patients from these EDs might help to reduce delays and improve access for more acute patients [3,4]. According to the present study, however, screening patients with a triage in a combined ED does not reduce visits in tertiary acute services. There are studies [12,15] suggesting that neither waiting times nor delays are directly correlated with resources or demand, but rather with how smoothly the processes of working are in an ED. Additional studies are needed to characterize the patients who visit tertiary care EDs without a pre-check by the primary health care in order to improve patient flow in an ED of the kind described in this study.

The number of visits to primary care doctors during office hours was unchanged during 2003-2005 in both Vantaa and Espoo (Figure 2). Thus, the decrease in the patient visits to the GPs of Peijas ED did not cause an overflow of patients in the office hour general practice. There seems to be no extra work load for the daytime doctor services. Our results are in line with the suggestion that EDs also have customers of their own and that those patients are not likely to use ordinary day time services of the primary health care system [4].

The change in the number of visits to the private sector GPs was similar in Vantaa, where the triage was applied, as compared with the control community Espoo (Figure 3). There is evidence that there is a correlation between public and private sectors with respect to the need of health care and health care utilization [16]. If the demand for health care is considered to be unsatisfactory in the public sphere patients look for care in private sector institutes [17]. No such shift was seen in the current study. Even though the access for non-immediate patients to Peijas' combined ED was made more inconvenient by using ABCDE triage, the patients in Vantaa did not seek help from the private sector more often than those who had unlimited access to the ED in the control city (Espoo). Actually, the use of private sector GPs was more frequent in Espoo where no ABCDE-triage was applied.

Patient safety issues are important when applying triage in an ED. The key player in the present triage model is the nurse who makes the initial assessment of the patient upon arrival. In our previous report, no extra false diagnoses or complications were observed when non-urgent patients were allocated to the slowest triage group (waiting up to 5-6 hours at worst times [10]). This agrees with the finding that in many EDs around the world triage has been successfully run by experienced nurses [11,13,18]. Furthermore, there are reports suggesting that some activities formerly performed by physicians in primary health care were safely performed by trained nurses [19]. The quality of triage must be continuously monitored and the number of incorrect assessments minimized. Right now further studies are ongoing on the safety of the present triage system and on the waiting time changes induced by it. Preliminary data from Vantaa seems promising in safety issues [10] but more detailed studies must be carried out. In this process the leaders are key persons responsible for the sustainability of implemented changes [20]. In our experience, their efforts to maintain the triage system are essential for a successfully functioning system. This includes continuous follow-up of security parameters and feedback to the staff [20].

## Conclusion

We conclude that the ABCDE-triage may reduce the use of a primary health care ED. Triage may be associated with a slight increase in the work load in the emergency systems of tertiary health care but it does not seem to increase the work load during office hours of the public primary health care system. Neither does it automatically redirect patients to the private sector.

## Abbreviations

ED: Emergency department; GP: General practitioner

## Competing interests

The authors declare that they have no competing interests.

## Authors' contributions

JaK led and performed the intervention and wrote the manuscript. JoK and JM arranged the data from the tertiary health care, JoK also wrote the manuscript. RM arranged the data from the control city Espoo. MM arranged the data from the Peijas ED and Vantaa city. MC wrote the manuscript. TK arranged the data from private sector, analyzed the data and wrote the manuscript. All the authors have read and approved the final manuscript.

## Pre-publication history

The pre-publication history for this paper can be accessed here:

http://www.biomedcentral.com/1471-227X/10/12/prepub
